# Association between Pet Ownership and Threatened Abortion in Pregnant Women: The China Birth Cohort Study

**DOI:** 10.3390/ijerph192316374

**Published:** 2022-12-06

**Authors:** Zheng Zhang, Yunjiang Yu, Boyi Yang, Wenzhong Huang, Yunting Zhang, Yana Luo, Michael S. Bloom, Zhengmin Qian, Lauren D. Arnold, Rienna Boyd, Qingqing Wu, Ruixia Liu, Guanghui Dong, Chenghong Yin, The China Birth Cohort Study (CBCS) Group

**Affiliations:** 1 Department of Central Laboratory, Beijing Obstetrics and Gynecology Hospital, Capital Medical University, Beijing Maternal and Child Health Care Hospital, Beijing 100026, China; 2 Guangdong Provincial Engineering Technology Research Center of Environmental and Health Risk Assessment, Department of Occupational and Environmental Health, School of Public Health, Sun Yat-sen University, Guangzhou 510080, China; 3 State Environmental Protection Key Laboratory of Environmental Pollution Health Risk Assessment, South China Institute of Environmental Sciences, Ministry of Environmental Protection, Guangzhou 510655, China; 4 Department of Epidemiology and Preventive Medicine, School of Public Health and Preventive Medicine, Monash University, Melbourne, VIC 3004, Australia; 5 Department of Global and Community Health, George Mason University, Fairfax, VA 22030, USA; 6 Department of Epidemiology and Biostatistics, College for Public Health and Social Justice, Saint Louis University, 3545 Lafayette Avenue, Saint Louis, MO 63104, USA

**Keywords:** domestic pet, threatened abortion, pregnancy, birth cohort study

## Abstract

Background: The aim of this study was to assess the association between pet ownership and threatened abortion (TA) in pregnant Chinese women. Materials and Methods: We enrolled pregnant women from 18 provinces and autonomous regions across China between November 2017 and December 2020. Participants were grouped based on the presence or absence of pet ownership. Pet owners were further sub-grouped based on the presence or absence of close contact with their pets. Pet species included cats, dogs, and both. Generalised linear mixed models, with province as a random effect, were used to estimate the associations between pet ownership and TA. Results: Pet ownership, whether or not one had close contact with pets, was associated with greater odds of TA (OR: 1.30, 95% CI: 1.21, 1.40). Keeping pet cats (OR: 1.24, 95% CI: 1.11, 1.40), dogs (OR: 1.29, 95% CI: 1.18, 1.41), or both cats and dogs (OR: 1.36, 95% CI: 1.04, 1.68) during pregnancy were all risk factors for TA. We observed significant group differences (*p* for difference < 0.05) in pre-pregnancy body mass index, education levels, and annual household income. Conclusions: Cat or dog ownership during pregnancy was associated with an increased risk of TA, especially among overweight, less educated, or lower-income participants.

## 1. Introduction

Threatened abortion (TA) is defined as vaginal bleeding, a closed cervix, and the presence of a foetal heartbeat [1]. It is one of the most common complications in early pregnancy, with an incidence of approximately 25%, and can significantly increase the risk of several adverse pregnancy outcomes, such as abortion, preterm birth, intrauterine growth retardation, placenta previa, and low birth weight [2,3,4]. The challenges presented by the TA in China are even greater with the implementation of the three-child policy [5]. Because the etiology is complex and the predictive methods are unreliable, TA is difficult to predict [6]. Worse still, the efficiency of common treatments (hormone supplements) remains a subject of debate; thus, no effective therapy for TA is currently available [1]. Many factors, including heart disease, diabetes, hypertension, thyroid disease, reproductive tract disease, periodontal disease, hepatitis B, multiple pregnancies, assisted reproductive technology, a history of abortion, excessive gestational weight gain, the number of pregnancies, and polycystic ovary syndrome, have been reported as being associated with TA or spontaneous abortion [7,8,9,10,11,12]. However, some potentially important environmental risk factors for TA were not demonstrated, although cigarette smoking and alcohol consumption were studied [13,14]. From the perspective of public health, identifying modifiable environmental risk factors is an effective method for reducing the impact of TA.

Pet ownership has become substantially more popular in recent decades, with approximately 30–40% of families in China reported to own a pet [15]. However, the associations between pet ownership and health outcomes have not been consistent, and the underlying mechanisms remain unclear [16]. A prospective cohort study involving 709 pregnant women suggested that women who had cats or dogs at home during pregnancy had different vaginal flora and that the presence of a cat increased the frequency of self-reported urinary tract infections [17]. Ascending infections from the vagina into the uterine cavity can seriously compromise the pregnancy [18,19]. Domestic pets can also leave their owners, especially those in close contact with pets, at increased risk of bacterial or parasitic infections [20], some of which (e.g., *Helicobacter pylori*) may affect the development of spontaneous abortion [21]. Additionally, an experimental study suggested that gazing behaviour from dogs increased urinary oxytocin concentrations in owners, which is typically interpreted as calming and stress-reducing [22]. Oxytocin is also involved in the initiation and promotion of uterine contractions during delivery. As the uterus continuously expresses oxytocin receptors during pregnancy, exposure to pets may increase the risk of TA. These findings suggest that pet ownership is likely associated with TA. However, only a few studies have examined this association.

The current study aimed to investigate the associations between pet ownership and TA, emphasizing different pet species and their close contact with pets. We also identified susceptible sub-group populations using data collected from the China Birth Cohort Study (CBCS), a nationwide study involving pregnant women recruited from 41 research sites across 18 provinces and autonomous regions in China.

## 2. Materials and Methods

### 2.1. Study Population

The CBCS is a multistage, multicentre, prospective, longitudinal, mega-cohort study, and national-based birth cohort study performed between November 2017 and December 2021. Pregnant women in the CBCS were recruited from 41 research sites across 18 provinces and autonomous regions in China [23]. The inclusion criteria were as follows: (1) Chinese nationality and 6–13 weeks of gestation; (2) planning to undergo routine antenatal examination, deliver at the study site, and continue to live locally for over 1 year; (3) no notifiable infectious diseases, such as hepatitis B, syphilis, and HIV; and (4) being able to understand the study and willing to provide valid informed consent. Women can withdraw from the study at any stage. Participants enrolled in the CBCS from November 2017 to December 2020 were included in our study, excluding those lost to follow-up and those without complete information on TA diagnosis, pet exposures, and confounders.

Participants were interviewed face-to-face by certified obstetricians, gynaecologists, or nurses during each of the following periods: 6–13, 20–23, and 28–33 weeks of gestation. Standard questionnaires were used during the interview to obtain information on demographic characteristics, reproductive history, medical history, family history, medical therapy, pet exposure history, toxicant exposure history, and lifestyle. The study was approved by the appropriate ethics committees. Written informed consent was obtained from all participants. All procedures contributing to this work complied with the ethical standards of the relevant national and institutional committees on human experimentation and with the Code of Ethics of the World Medical Association (Declaration of Helsinki) for experiments involving humans.

### 2.2. Diagnosis of Threatened Abortion

TA is defined as vaginal bleeding with or without abdominal pain while the cervix is closed and the foetus is viable and inside the uterine cavity [24]. TA was diagnosed before 20 weeks of gestation [25] by certified obstetricians and gynecologists who rigorously followed the above definition, and it was recorded in medical records. TA, which developed before enrolment, was diagnosed at enrolment. Those who developed TA after the first interview but before the 20 weeks of gestation were diagnosed at the second interview.

### 2.3. Assessment of Pet Ownership

Information regarding pet exposure was collected upon enrolment during the 6–13 weeks of gestation. Pet ownership was assigned according to participants’ answers to three questions. Question 1: Did you keep any domestic pets during pregnancy? Question 2: What pet did you keep? Question 3: Did you often have close contact (petting, grooming, cleaning, feeding, playing, and kissing [26]) with your pet? Participants could answer with “yes”, “no”, or “I don’t want to answer” to question 1. Participants who refused to answer were considered lost for follow-up. If participants answered yes to question 1, they were then asked questions 2 and 3. Participants who kept at least one indoor pet were also subdivided into the following two groups: Group 1, pet owners without close contact with pets; Group 2, close contact with pets. Pet species were divided into three categories. Participants who kept at least one dog but no cats, with or without any other domestic pets; participants who kept at least one cat but no dogs, with or without any other domestic pets; participants who kept both dogs and cats, with or without any other domestic pets.

Participants who kept pets other than dogs or cats were treated as having no pet ownership for the following reasons: First, cats and dogs are two of the most popular domestic pets, and they have significantly closer relationships with humans than other pets. Second, most pet cats and dogs have free access to their owners’ main activities, such as living rooms and bedrooms, while other pets are mostly kept in cages or fish tanks and have significantly smaller home ranges than cats and dogs.

### 2.4. Covariates

Based on the existing literature, we selected important covariates, including potential confounders, using the following three criteria: (1) possible risk factors for TA; (2) antecedent to pet exposure (i.e., a ‘cause’ of pet exposure) and unequal distribution between exposed and unexposed groups; and (3) not an ‘effect’ of pet exposure or an intermediate factor in the causal pathway of TA [27]. We retained the following covariates in our final models based on published literature: average daily working time (hours), ethnicity (Han and minority ethnicities), education levels (below university, university, and above), household income (CNY <200,000 and ≥200,000 per year), and house types (high-rise apartment buildings and ‘others’, such as single-story, single-family, or separated residences).

### 2.5. Statistical Analysis

The Shapiro–Wilk test was used to assess the normality of quantitative variables. The Chi-square test and Student’s *t*-test were used to investigate baseline differences between demographic variables and TA. Continuous variables were presented as means and standard deviations, whereas categorical data were described as frequencies and percentages.

Generalised linear mixed-effects regressions were used to estimate associations between pet exposure and TA, in which the province was used as a random effect. We applied two regression models: (1) a crude model without adjustment and (2) an adjusted model (adjusted for confounding by average daily working time, ethnicity, education levels, house types, and annual household income). We then analysed the associations between pet ownership and TA by dividing pet owners into those without close contact with pets and those in close contact with pets. We also analysed the associations of cat and dog ownership with TA.

Stratified analyses were conducted to explore the potential effects of age (≥35 and <35 years), overweight or obesity (pre-pregnancy BMI < 24 and ≥24 kg/m^2^), education levels (below university and university and above), and household income (CNY <200,000 and ≥200,000/year). The analysis was repeated for each sub-group, after which *p* values for group differences were calculated by a two-sample *t*-test.

Sensitivity analyses were conducted to evaluate the robustness of our results by excluding the following participants: those who currently smoke or have smoked in the past; those who currently consume alcohol or have consumed alcohol in the past; those who had multiple pregnancies; those who had assisted reproductive technology; and those who have a history of abortion or pre-pregnancy diseases (defined as having at least one of the following diagnoses before pregnancy: heart disease, diabetes, hypertension, thyroid disease, reproductive tract disease, periodontal disease, or hepatitis B) [7,8,9]. To minimise provincial bias, the analysis was repeated after individually excluding each province or municipality. Additionally, women recruited from December 2019 onwards were excluded, considering the effect of pandemic-related stress and social isolation on TA. Furthermore, we adjusted the number of pregnancies in the main model to control for their potential confounding effect.

Statistical significance was tested using two-tailed hypothesis tests, with *p* values < 0.05 indicating statistical significance. All analyses were conducted using R (version 4.0.3, R Foundation for Statistical Computing).

## 3. Results

### 3.1. Population Characteristics

**Figure 1:**
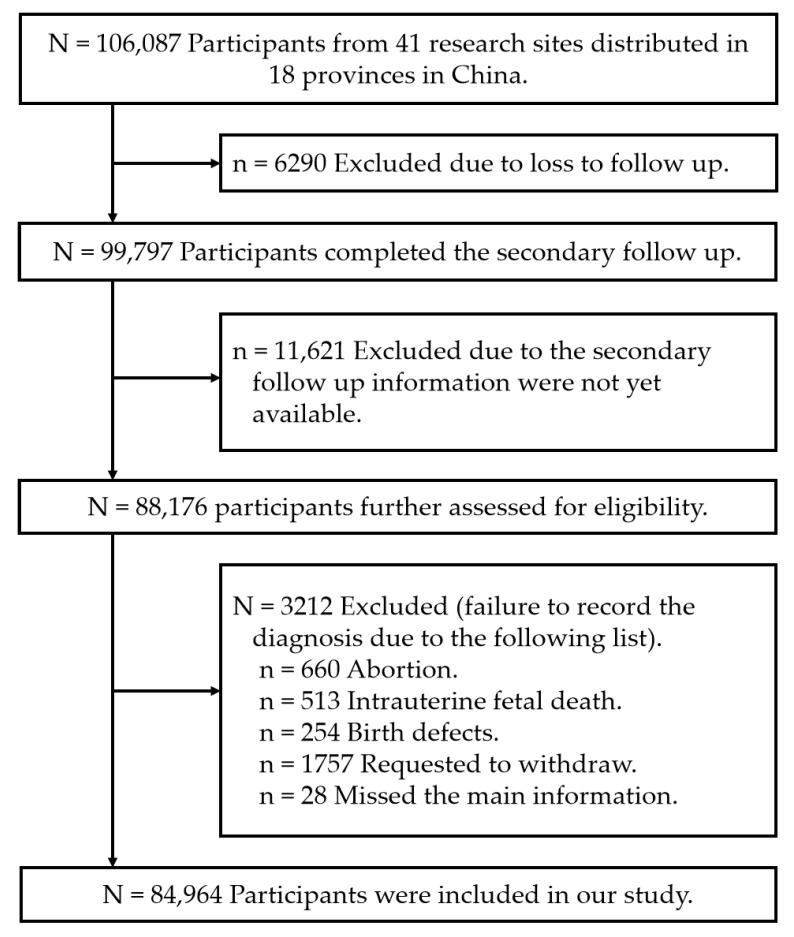
Flowchart of study enrolment, with the participants selected from 41 research sites distributed in 18 provinces or municipalities during November 2017 to December 2020 (n = 84,964).

Figure 1 shows the flowchart of study enrolment. Accordingly, 106,087 women with a pregnancy in the CBCS were enrolled, with 84,964 and 21,123 women being included and excluded, respectively. Most provinces of China were covered, and Figure S1 shows the geographical distribution of the participants. Table 1 shows that 10,549 (12.4%) participants were older than 35 years, and 8579 (10.10%) owned a pet cat or dog. Approximately 9% (n = 7379) of the participants developed TA, with the TA and non-TA groups showing differences in the distribution of pet ownership (13.2% vs. 9.9%, respectively). The distribution of sociodemographic, clinical, and pet exposure characteristics was similar for the CBCS participants before (n = 106,087) and after (n = 84,964) exclusion (Table S1).

### 3.2. Main Analyses

In the adjusted model, pet ownership was associated with a 1.30-fold (95% CI: 1.21, 1.40) increase in the odds of TA. Similar results were observed in the crude model. Pet owners without close contact with pets (OR: 1.25, 95% CI: 1.11, 1.40) and those in close contact with pets (OR: 1.36, 95% CI: 1.23, 1.50) were both significantly associated with increased odds of TA. Similarly, keeping domestic pet cats (OR: 1.24, 95% CI: 1.11, 1.40), dogs (OR: 1.29, 95% CI: 1.18, 1.41) or both (OR: 1.36, 95% CI: 1.04, 1.68) during pregnancy were all risk factors of TA (Table 2).

Sensitivity analyses suggest that the significance and direction of our results were robust and consistent when current or former smokers were excluded, when current or former drinkers were excluded, when those with multiple pregnancies were excluded, and when the assisted reproductive participants were excluded. We excluded all participants from a province one at a time and found the estimates had not changed substantially. The result was consistent with the main analysis when women recruited from December 2019 onwards were excluded. Furthermore, we observed a robust result when we additionally adjusted the number of pregnancies (Table S2).

### 3.3. Stratified Analyses

**Figure 2:**
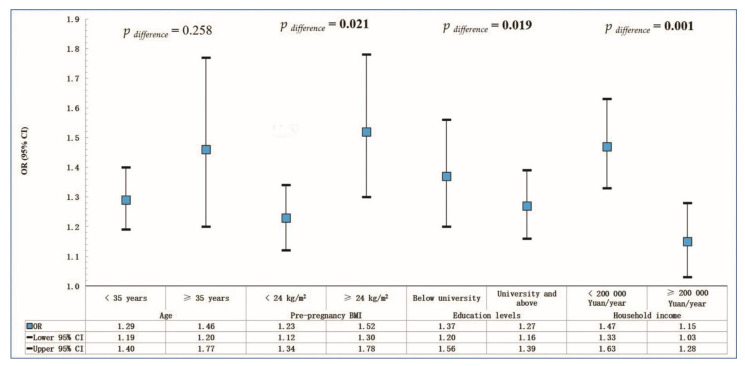
Adjusted odds ratios (ORs) and 95% confidence intervals (CIs) of associations between pet ownership and threatened abortion, stratified by potential modifiers. Adjusted for average daily working hours, ethnicity, educational levels, average annual household income, and house types.

Sub-group analyses showed that the associations between pet ownership and TA were stronger in those who were overweight or obese (*P*_difference_ = 0.021), had less than a university-level education (*P*_difference_ = 0.019), and had a household income of CNY <200,000 per year (*P*_difference_ = 0.001) compared to their counterparts (Figure 2). The modifying effects of the three factors on the association of pet ownership and TA remain whether or not there was close contact with pets (Table S3). We found a similar effect of modifications on the association between cat and dog ownership and TA (Table S4).

## 4. Discussion

### 4.1. Principal Findings

This is the first nationwide birth cohort study to report an association between pet ownership and TA. The results of our study suggest that keeping a pet cat or dog during pregnancy is associated with greater odds for TA than not keeping a pet cat or dog. Pet ownership, whether or not there was close contact with pets, was associated with an increased risk of TA. Keeping domestic pet cats, dogs, or both were all risk factors for TA. Stronger associations were observed among pregnant women who were overweight, had less than a university-level education, and had an annual household income of CNY <200,000.

### 4.2. Strengths and Limitations

Our study has several strengths. We used a very large national birth cohort and a prospective cohort study design to assess the relationship between pet ownership and TA in China, which helped ensure the internal validity of the findings. Clinically confirmed diagnoses were used to minimise outcome misclassification, and a face-to-face study questionnaire was delivered by health professionals to limit exposure and covariate misclassification. We conducted detailed sensitivity analyses to determine the robustness of the main model. However, several limitations of this study warrant discussion. First, the questionnaire used for enrolment included retrospective questions, which could potentially result in exposure misclassification. Second, no detailed information was available regarding the number of pets, which could affect the accuracy of the results; however, this will not affect the conclusions. Third, we have no laboratory data to verify this correlation, which should be considered in interpreting the causal strength. Fourth, the differences in TA diagnosis between the provinces remain, which may induce misclassification. Thus, we conducted several sensitivity analyses, and a generalised linear model with province as the random effect was used to minimise these differences. Fifth, pregnant women were at a higher risk of miscarriage during 6–13 weeks of gestation; thus, the miscarriage-related bias remains possible despite the exclusion of those who had a miscarriage. Finally, residual confounding remains a possibility due to unmeasured factors such as the assessment of depression, anxiety, stress, and physical activity, the per capita area of homes, and the availability of elevators at home and at the workplace.

### 4.3. Interpretation

Aside from our study, no evidence exists regarding the association between pet ownership and TA. A previous study including 100 participants reported that being overly intimate with a pet was significantly associated with abortion; however, the association did not reach significance in their adjusted model [28]. In contrast, the current study found positive associations between pet ownership and TA in both crude and adjusted models, whether or not there was close contact with pets. The reason for the difference between our results and those of the previous study may be mostly due to the sample size. Additionally, we could not preclude the possibility that the causal pathway driving the associations between pet ownership and TA differs from that driving the associations between pet ownership and spontaneous abortion.

Although the mechanisms underlying pet ownership and TA are not entirely clear, the following may offer some explanations: First, domestic pets leave their owners, especially those in close contact with pets, at an increased risk of bacterial or parasitic infections [20], some of which are able to pass the placental barrier and seriously compromise the pregnancy [18,19], and may further affect the development of spontaneous abortion [21]. Second, in some countries, the rate of human *T. gondii* infection ranges from 10% to over 50%, with cats being an important source [29]. *T. gondii* infection can cause placental inflammation, which is an important risk factor for spontaneous abortion [30]. Studies have also suggested that pet ownership increases the risk of *Listeria* infection [31]. Owing to the decreased immune level of women during pregnancy, the unrestricted proliferation of *Listeria* in the liver, which is the first target organ after intestinal translocation, may result in prolonged low-level bacteremia, leading to an invasion of the gravid uterus, which is the preferred secondary target organ [32]. Third, owners are often required to crouch or bend down to clean or bathe their pets, which has been reported to increase the risk of abortion during pregnancy [33,34]. Fourth, caring for an extra family member (pet cats or dogs) may increase stress in pregnant women. A recent study found that, contrary to normal expectations, dog owners were more stressed than non-dog owners [35]. However, stress has also been reported as a risk factor for spontaneous abortion [36,37]. Finally, domestic pets are a major source of allergens and house dust endotoxins [38]. Despite limited evidence from human populations, animal studies have indicated a link between endotoxin exposure and adverse pregnancy outcomes [39,40]. Most importantly, pet exposure may increase oxytocin concentrations in owners and alter the gut flora, which may further cause TA [22,41].

Notably, zoonotic hazards are reported to rarely accompany pet ownership [42]; therefore, domestic pets are unlikely to increase TA risk through zoonosis. Furthermore, the generalisability of results from our study may be limited due to the unmeasured residual confounding variables. Despite citing a few potential explanations for our results, our conclusions should be interpreted with caution. Future studies should assess the duration of pet exposure, determine the number of domestic pets, and include more potential confounding factors and details of domestic pets (such as more pet species and the pet’s age, gender, and health conditions) to confirm our findings. More concrete studies are needed to determine the underlying molecular mechanisms.

Our findings showed that keeping a domestic pet cat or dog during pregnancy was associated with an increased risk of TA. Furthermore, pet owners in close contact with their pets had a slightly higher risk of TA than those without close contact. This could be partially explained by the fact that close contact with pets promotes more active disease transmission, such as *T. gondii* infection [43]. Our study showed that either dog or cat ownership during pregnancy could increase the risk of TA. On the one hand, cat allergens, rather than dog allergens, can induce the production of tumour necrosis factor and interleukin-6 [44], which are inflammatory cytokines associated with adverse pregnancy outcomes [45]. Another study showed that the gazing behaviour of dogs increased urinary oxytocin concentrations in owners; cats were not mentioned in this study [22]. On the other hand, both cats and dogs can increase the concentrations of indoor allergens and house dust endotoxins [38] and alter the gut flora [41,46]. Collectively, exposure to domestic pet cats and dogs may involve both common and unique pathogenic mechanisms.

Our results suggest that the association between pet ownership and TA among overweight or obese pregnant women is stronger than that in the normal population. A Chinese prospective cohort study found that obesity significantly increased the risk of spontaneous abortion [47], and our results further support an interaction effect between pet ownership and being overweight. Previous studies have shown that awareness of zoonotic and invasive fungal diseases is highly correlated with education levels [48,49]. Accordingly, increased awareness of the risk factors for a condition encourages the adoption of precautionary measures, thereby lowering the chances of being affected. These findings support our results, which showed that women with a low level of education had higher odds of TA when exposed to a domestic pet. A higher average annual household income may indicate better indoor hygiene and lower the risk of infection from pet ownership [50].

## 5. Conclusions

Our study suggests that pet ownership, with or without close contact with pets, was significantly associated with increased odds of TA, especially for those who were overweight, had less than a university-level education, or had an annual household income of CNY <200,000. Future research should look into other populations and focus on the use of appropriate study designs and improved exposure assessment to confirm our findings.

## Figures and Tables

**Table 1:** Characteristics of study participants (n = 84,964).

Variables	Total	TA	None-TA	*p* Value ^1^
	(n = 84,964)	(n = 7379)	(n = 77,585)	
Age (years), mean (SD)	29.89 (4.22)	30.76 (4.17)	29.81 (4.21)	<0.001
<35 years	74,415 (87.6)	6124 (83.0)	68,291 (88.0)	
≥35 years	10,549 (12.4)	1255 (17.0)	9294 (12.0)	
Pre-pregnancy BMI (≥24 kg/m ^2^)	17,495 (20.6)	1502 (20.4)	15,993 (20.6)	0.379
Daily working time	7.78 (2.19)	7.87 (2.22)	7.78 (2.18)	0.001
(hours), mean (SD)				
Primigravida (Yes)	41,567 (48.9)	3507 (47.5)	38,060 (49.1)	0.012
Pre-pregnancy diseases (Yes)	7358 (8.7)	907 (12.3)	6451 (8.3)	<0.001
History of abortion (Yes)	25,953 (30.5)	2769 (37.5)	23,184 (29.9)	<0.001
Ethnicity				<0.001
Han	79,735 (93.8)	6798 (92.1)	72,937 (94.0)	
Minority ethnicities	5229 (6.2)	581 (7.9)	4648 (6.0)	
Education levels				<0.001
University and above	43,775 (51.5)	4614 (62.5)	39,161 (50.5)	
Below university	41,189 (48.5)	2765 (37.5)	38,424 (49.5)	
Annual household income				<0.001
CNY ≥200,000	26,304 (31.0)	3258 (44.2)	23,046 (29.7)	
CNY <200,000	58,660 (69.0)	4121 (55.8)	54,539 (70.3)	
Housing types				<0.001
High-rise apartment buildings	74,232 (87.4)	6742 (91.4)	67,490 (87.0)	
Others ^3^	10,732 (12.6)	637 (8.6)	10,095 (13.0)	
Singleton pregnancy (Yes)	83,376 (98.1)	7180 (97.3)	76,196 (98.2)	<0.001
Conception ways				<0.001
Normal pregnancy	80,981 (95.3)	6804 (92.2)	74,177 (95.6)	
Assisted reproductive technology ^4^	3983 (4.7)	575 (7.8)	3408 (4.4)	
Cigarette smoking				<0.001
Current or former	1956 (2.3)	255 (3.5)	1701 (2.2)	
Never	83,008 (97.7)	7124 (96.5)	75,884 (97.8)	
Alcohol consumption				0.002
Current or former	2736 (3.2)	282 (3.8)	2454 (3.2)	
Never	82,228 (96.8)	7097 (96.2)	75,131 (96.8)	
Pet species				<0.001
No pet ownership	76,385 (89.9)	6407 (86.8)	69,978 (90.1)	
Cat only	2537 (3.0)	303 (4.1)	2234 (2.9)	
Dog only	5466 (6.4)	605 (8.2)	4861 (6.3)	
Both cats and dogs	576 (0.7)	64 (0.9)	512 (0.7)	
Pet exposure				<0.001
No pet ownership	76,385 (89.9)	6407 (86.8)	69,978 (90.1)	
Pet owners without close contact with pets	2780 (3.3)	287 (3.9)	2493 (3.2)	
In close contact with pets	5799 (6.8)	685 (9.3)	5114 (6.6)	

Note: Values are presented as n (%) except where indicated; if someone only kept farm animals or other pets but not a dog or cat, they will be considered to have no pet exposure in this study. Abbreviations: TA: threatened abortion; BMI: body mass index; SD: standard deviation. ^1^ According to the χ^2^ test for nominal variables and the *t*-test for continuous variables, there is a significant difference (*p* < 0.05) across TA conditions; ^2^ Pre-pregnancy diseases included heart diseases, diabetes, hypertension, thyroid diseases, reproductive tract diseases, periodontal diseases, and hepatitis B; ^3^ Single-story, single-family, separated residences, or other houses that are not high-rise buildings; ^4.^ Assisted reproductive technology included in-vitro fertilization and artificial insemination.

**Table 2:** Odds ratios (ORs) and 95% confidence intervals (CIs) of associations between pet exposure and threatened abortion.

	Crude Model		Adjusted Model ^1^	
	OR (95%CI)	*p* Value	OR (95%CI)	*p* Value
Pet ownership	1.42 (1.33, 1.52)	<0.001	1.30 (1.21, 1.40)	<0.001
Pet owners without close contact with pets	1.35 (1.22–1.49)	<0.001	1.25 (1.11, 1.40)	<0.001
In close contact with pets	1.52 (1.39, 1.67)	<0.001	1.36 (1.23, 1.50)	<0.001
Pet species				
Cat only	1.43 (1.28, 1.59)	<0.001	1.24 (1.11, 1.40)	<0.001
Dog only	1.36 (1.25, 1.47)	<0.001	1.29 (1.18, 1.41)	<0.001
Both cats and dogs	1.31 (1.11, 1.53)	0.001	1.36 (1.04, 1.68)	<0.001

^1^ Adjusted for average daily working hours, ethnicity, educational levels, average annual household income, and house types.

## Data Availability

The data presented in this study are available on request from the corresponding author. The data are not publicly available due to the privacy of pregnant women’s data, which is respected and enforced.

## Supplementary Materials

The following supporting information can be downloaded at: https://www.mdpi.com/article/10.3390/ijerph192316374/s1, Figure S1: Participants’ residential location in the present study (n = 84,964); Table S1: Characteristics of all (n = 106,087), included (n = 84,964), and excluded (n = 21,123) participants; Table S2: The adjusted ORs and 95%CIs of pet ownership and threatened abortion after excluding special participants; Table S3: Associations of pet owners without close contact with pets and in close contact with pets on threatened abortion, stratified by potential modifiers; Table S4: Associations of cat and dog ownership with threatened abortion, stratified by potential modifiers.

## Appendix A

There were more than 400 members in the China Birth Cohort Study (CBCS) Group as of 2020. Among whom were Wen-Tao Yue, Yue Zhang, Shen Gao, En-Jie Zhang, Shao-Fei Su, Xiao Gao, Jian-Hui Liu, Shuang-Hua Xie, Hao Xing, Ying-Yi Luan, and Tian-He Li, who were listed as co-authors of this study. All of them come from the Department of Central Laboratory, Beijing Obstetrics and Gynecology Hospital, and Capital Medical University.
