# Supplementary material for: Association between Pet Ownership and Threatened Abortion in Pregnant Women: The China Birth Cohort Study
Source: Int J Environ Res Public Health. 2022 Dec 6;19(23):16374. doi: 10.3390/ijerph192316374 (PMC9739756; doi:10.3390/ijerph192316374)
Supplement: Supplementary file 1 [file ijerph-19-16374-s001.zip › ijerph-2012327-supplementary.pdf]

Supplemental Material

Associations between pet ownership and threatened abortion: The China Birth Cohort Study

Table of Contents

Table S1. Characteristics of all (n = 106,087), included (n = 84,964), and excluded (n = 21,123) participants.

Table S2. The adjusted ORs and 95%CIs of pet ownership and threatened abortion after excluding special participants.

Table S3. Associations of pet owners without close contact with pets and in close contact with pets on threatened abortion, stratified by potential modifiers.

Table S4. Associations of cat ownership and dog ownership with threatened abortion, stratified by potential modifiers.

Figure S1. Participants' residential location in the present study (n = 84,964)

Table S1. Characteristics of all (n = 106,087), included (n= 84 964), and excluded (n= 21,123) participants.

Variables	All participants (n = 106,087)	Participants included in the present analysis (n = 84,964)	Participants excluded from the present analysis (n = 21,123)
Age (≥ 35 years)	13,367 (12.6)	10,549 (12.4)	2,818 (13.3)
Pre-pregnancy BMI (≥ 24 kg/m^2)	21,960 (20.7)	17,495 (20.6)	4,465 (21.1)
Daily working time (Hours), mean (SD)	7.80 (2.36)	7.78 (2.19)	7.88 (2.55)
Primigravida (Yes)	51,558 (48.6)	41,567 (48.9)	9,991 (47.3)
Pre-pregnancy diseases [1] (Yes)	9,230 (8.7)	7,358 (8.7)	1,872 (8.9)
History of abortion (Yes)	32,781 (30.9)	25,953 (30.5)	6,828 (32.2)
Ethnicity			
Han	99,191 (93.5)	79,735 (93.8)	19,456 (92.1)
Minority ethnicities	6,896 (6.5)	5,229 (6.2)	1,667 (7.9)
Education levels			
University and above	54,741 (51.6)	43,775 (51.5)	10,966 (51.9)
Below university	51,346 (48.4)	41,189 (48.5)	10,157 (48.1)
Average household income			
≥ 200, 000 yuan/year	32,463 (30.6)	26,304 (31.0)	6,159 (29.2)
< 200 000 yuan/year	73,624 (69.4)	58,660 (69.0)	14,964 (70.8)
Housing types			
High-rise apartment buildings	92,402 (87.1)	74,232 (87.4)	18,170 (86.0)
Others [2]	13,685 (12.9)	10,732 (12.6)	2,953 (14.0)
Singleton pregnancy (Yes)	103,753 (97.8)	83,376 (98.1)	20,377 (96.5)
Conception ways			
Normal pregnancy	101,101 (95.3)	80,981 (95.3)	20,120 (95.3)
Assisted reproductive technology [3]	4,986 (4.7)	3,983 (4.7)	1,427 (4.7)
Cigarette smoking			
Current or former	2,440 (2.3)	1,956 (2.3)	484 (2.3)
Never	103,647 (97.7)	83,008 (97.7)	20,639 (97.7)
Alcohol consumption			
Current or former	3,395 (3.2)	2,736 (3.2)	659 (3.1)
Never	102,692 (96.8)	82,228 (96.8)	20,464 (96.9)
Pet species			
No pet ownership	95,481 (90.0)	76,385 (89.9)	19,096 (90.4)
Cat only	3,180 (3.0)	2,537 (3.0)	643 (3.0)
Dog only	6,683 (6.3)	5,466 (6.4)	1,217 (5.8)
Both cat and dog	743 (0.7)	576 (0.7)	167 (0.8)
Pet exposure			

No pet ownership	95,481 (90.0)	76,385 (89.9)	19,096 (90.4)
Pet owners without close contact with pets	3,498 (3.3)	2,780 (3.3)	718 (3.4)
Close contact with pets	7,108 (6.7)	5,799 (6.8)	1,309 (6.2)

Note: Values are presented as n (%) except where indicated.

[1] Pre-pregnancy diseases include heart diseases, diabetes, hypertension, thyroid diseases, reproductive tract diseases, periodontal diseases, and hepatitis B;

[2] Single-story, single-family, separated residences, or other houses which are not high-rise buildings;

[3] Assisted reproductive technology included in-vitro fertilization and artificial insemination.

Table S2. The adjusted ORs and 95%CIs of pet ownership and threatened abortion after excluding special participants.

**Models**	**n**	**Pet ownership**	
		**OR (95%CI)**	***P* value**
After excluding cigarette smoking participants	83,008	**1.28 (1.19, 1.39)**	**< 0.001**
After excluding alcohol consumption participants	82,228	**1.31 (1.21, 1.41)**	**< 0.001**
After excluding multiple pregnancies participants	83,376	**1.36 (1.25, 1.48)**	**< 0.001**
After excluding assisted reproductive participants	80,981	**1.26 (1.09. 1.42)**	**< 0.001**
After excluding participants with history of abortion	59,011	**1.31 (1.20, 1.43)**	**< 0.001**
After excluding participants with pre-pregnancy diseases	77,606	**1.29 (1.19, 1.40)**	**< 0.001**
After excluding participants from Beijing	58,989	**1.14 (1.02, 1.35)**	**0.046**
After excluding participants from Guangdong	74,397	**1.25 (1.16, 1.35)**	**< 0.001**
After excluding participants from Hainan	81,703	**1.30 (1.20, 1.40)**	**< 0.001**
After excluding participants from Hebei	82,723	**1.30 (1.21, 1.40)**	**< 0.001**
After excluding participants from Henan	79,901	**1.30 (1.20, 1.40)**	**< 0.001**
After excluding participants from Hunan	81,611	**1.30 (1.21, 1.40)**	**< 0.001**
After excluding participants from Jiangsu	83,481	**1.31 (1.22, 1.41)**	**< 0.001**
After excluding participants from Jiangxi	82,830	**1.30 (1.20, 1.40)**	**< 0.001**
After excluding participants from Liaoning	83,147	**1.31 (1.21, 1.41)**	**< 0.001**
After excluding participants from Inner Mongolia	83,248	**1.30 (1.20, 1.40)**	**< 0.001**
After excluding participants from Shandong	69,167	**1.22 (1.13, 1.32)**	**< 0.001**
After excluding participants from Sichuan	79,260	**1.33 (1.23, 1.43)**	**< 0.001**
After excluding participants from Tianjin	84,456	**1.30 (1.21, 1.40)**	**< 0.001**
After excluding participants from Yunnan	79,653	**1.34 (1.24, 1.44)**	**< 0.001**
After excluding participants from Gansu	84,944	**1.31 (1.21, 1.41)**	**< 0.001**
After excluding participants from Guizhou	84,958	**1.31 (1.21, 1.41)**	**< 0.001**
After excluding participants from Shaanxi	84,958	**1.31 (1.21, 1.41)**	**< 0.001**
After excluding participants from Jilin	84,959	**1.31 (1.21, 1.41)**	**< 0.001**
After excluding participants recruited from December 1st 2019 onwards	52,584	**1.29 (1.19, 1.39)**	**< 0.001**
Additionally adjusted for number of pregnancies	84,964	**1.30 (1.20, 1.41)**	**< 0.001**

Adjusted for average daily working hours, ethnicity, educational levels, average annual household income and house types.

Table S3. Associations of pet owners without close contact with pets and in close contact with pets on threatened abortion, stratified by potential modifiers.

Variables	n	Pet exposures (OR, 95%CI, $P_{difference}$)					
		**Pet owners without close contact with pets**	***P*** [1]	**Close contact with pets**	***P*** [1]	**Pet ownership** [2]	***P*** [1]
Age			0.459		0.110		0.258
< 35 years	74,415	1.13 (0.87, 1.31)		1.33 (1.19, 1.48)		1.29 (1.19, 1.40)	
≥ 35 years	10,549	1.28 (1.14, 1.44)		1.67 (1.28, 2.14)		1.46 (1.20, 1.77)	
Pre-pregnancy BMI			**0.032**		**0.008**		**0.021**
< 24 kg/m^2	67,469	1.17 (1.01, 1.34)		1.27 (1.13, 1.43)		1.23 (1.12, 1.34)	
≥ 24 kg/m^2	17,495	1.35 (1.15, 1.50)		1.77 (1.43, 2.17)		1.52 (1.30, 1.78)	
Education levels			**0.027**		**0.038**		**0.019**
University and above	43,775	1.25 (1.09, 1.43)		1.27 (1.13, 1.43)		1.27 (1.16, 1.39)	
Below university	41,189	1.44 (1.21, 1.76)		1.60 (1.33, 1.91)		1.37 (1.20, 1.56)	
Household income			**0.030**		**0.006**		**0.001**
≥ 200 000 Yuan/year	26,304	1.14 (1.02-1.27)		1.13 (1.01-1.26)		1.15 (1.03, 1.28)	
< 200 000 Yuan/year	58,660	1.40 (1.20, 1.62)		1.58 (1.36, 1.81)		1.47 (1.33, 1.63)	

Adjusted for average daily working hours, ethnicity, educational levels, average annual household income and house types, except for sometimes when they are used as the grouping variable.

[1] P difference < 0.05 suggests significant different OR values between the two subgroups;

[2] Pet ownership is a combination of pet owners without close contact with pets and close contact with pets.

Table S4. Associations of cat ownership and dog ownership with threatened abortion, stratified by potential modifiers.

Variables	n	Pet species (OR, 95%CI, $P_{difference}$)							
		**Cats**	***P*** [1]	**Dogs**	***P*** [1]	**Both cat and dog**	***P*** [1]	**Pet ownership** [2]	***P*** [1]
Age			0.826		0.154		0.255		0.258
< 35 years	74,415	1.26 (1.09, 1.45)		1.29 (1.15, 1.43)		1.41 (1.02, 1.91)		1.29 (1.19, 1.40)	
≥ 35 years	10,549	1.42 (1.18, 1.86)		1.58 (1.21, 2.05)		0.75 (0.22, 1.89)		1.46 (1.20, 1.77)	
Pre-pregnancy BMI			**0.039**		**0.023**		0.301		**0.021**
< 24 kg/m^2	67,469	1.14 (1.02, 1.34)		1.21 (1.07, 1.37)		1.45 (1.01, 2.01)		1.23 (1.12, 1.34)	
≥ 24 kg/m^2	17,495	1.56 (1.15, 2.07)		1.60 (1.30, 1.95)		0.89 (0.34, 1.92)		1.52 (1.30, 1.78)	
Education levels			**0.021**		**0.016**		0.552		**0.019**
University and above	43,775	1.27 (1.09, 1.47)		1.24 (1.09, 1.40)		1.22 (0.82, 1.76)		1.27 (1.16, 1.39)	
Below university	41,189	1.48 (1.27, 1.78)		1.44 (1.21, 1.69)		1.24 (0.57, 2.34)		1.37 (1.20, 1.56)	
Household income			**0.003**		**0.005**		0.086		**0.001**
≥ 200 000 Yuan/year	26,304	1.18 (1.09, 1.44)		1.12 (1.01, 1.29)		0.98 (0.59, 1.53)		1.15 (1.03, 1.28)	
< 200 000 Yuan/year	58,660	1.36 (1.10, 1.67)		1.49 (1.30, 1.69)		1.67 (1.11, 2.42)		1.47 (1.33, 1.63)	

Adjusted for average daily working hours, ethnicity, educational levels, average annual household income and house types, except for sometimes when they are used as the grouping variable.

[1] $P_{difference} < 0.05$ suggests significant different OR values between the two subgroups;

[2] Pet ownership is a combination of cats, dogs and both cat and dog.

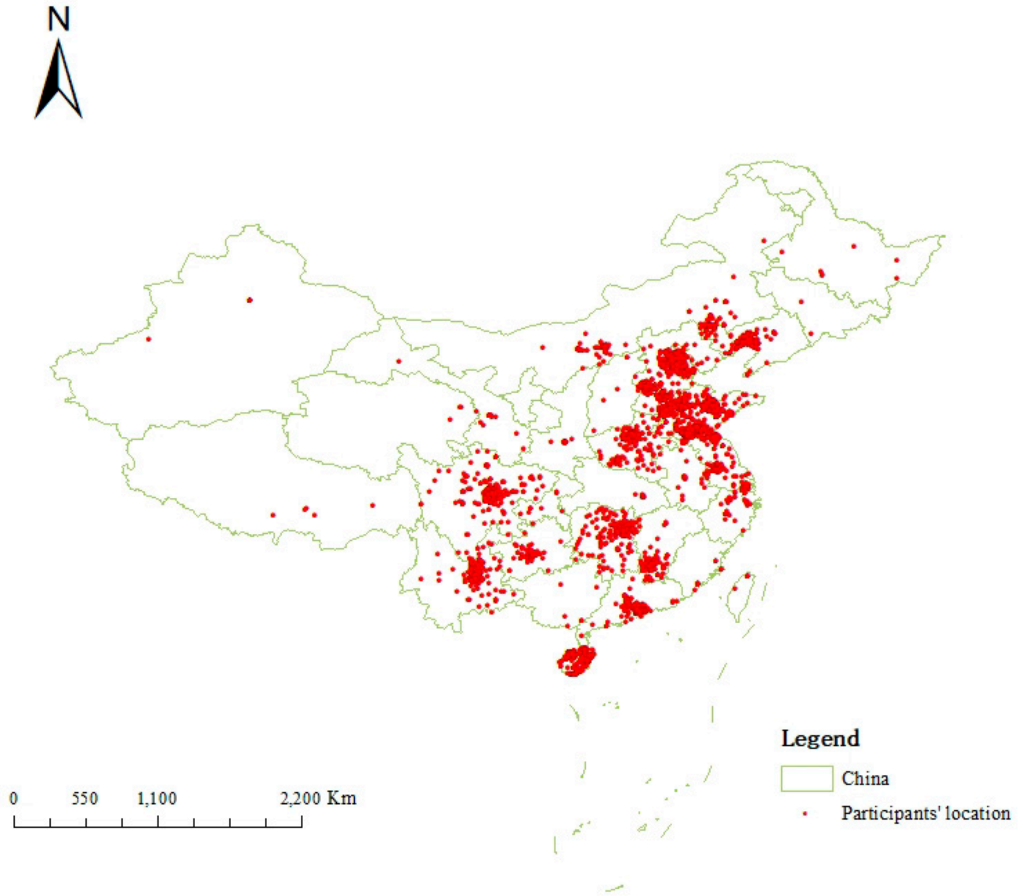

Figure S1. Participants' residential location in the present study (n = 84,964)
